# Single Nucleotide Polymorphisms in 25-Hydroxyvitamin D3 1-Alpha-Hydroxylase (*CYP27B1*) Gene: The Risk of Malignant Tumors and Other Chronic Diseases

**DOI:** 10.3390/nu12030801

**Published:** 2020-03-18

**Authors:** Maria Latacz, Jadwiga Snarska, Elżbieta Kostyra, Ewa Fiedorowicz, Huub F. J. Savelkoul, Roman Grzybowski, Anna Cieślińska

**Affiliations:** 1 Faculty of Biology and Biotechnology, University of Warmia and Mazury, 10-719 Olsztyn, Poland; mmlatacz@gmail.com (M.L.); elzbieta.kostyra@uwm.edu.pl (E.K.); ewa.kuzbida@uwm.edu.pl (E.F.); 2 Faculty of Medicine, Collegium Medicum, University of Warmia and Mazury in Olsztyn, 10-082 Olsztyn, Poland; 3 Department of General Surgery, Faculty of Medical Sciences, Collegium Medicum, University of Warmia and Mazury, 10-082 Olsztyn, Poland; jadwiga.snarska@uwm.edu.pl; 4 Independent Public Healthcare Center of Ministry of Internal Affairs and Administration with the Warmia and Mazury, Center of Oncology in Olsztyn, 10-228 Olsztyn, Poland; 5 Cell Biology and Immunology Group Wageningen University and Research, 6700 AH Wageningen, The Netherlands; huub.savelkoul@wur.nl; 6 Clinical Department of Trauma-Orthopedic Surgery and Spine Surgery of the Provincial Specialist Hospital in Olsztyn, 10-561 Olsztyn, Poland; romek.grzybowski@wp.pl; 7 Clinical Department of Orthopedics, Traumatology and Spine Surgery, Collegium Medicum, University of Warmia and Mazury, 10-719 Olsztyn, Poland

**Keywords:** vitamin D, single nucleotide polymorphism, CYP27B1, chronic diseases, colorectal cancer, breast cancer, prostate cancer, multiple sclerosis

## Abstract

Vitamin D is widely known for its roles in the promotion of apoptosis and differentiation, with simultaneous inhibition of proliferation, inflammation, angiogenesis, invasion, and metastasis. Modern literature lacks complete information on polymorphisms in *CYP27B1*, the only enzyme capable of vitamin D activation. This review presents gathered data that relate to genetic variants in *CYP27B1* gene in correlation to multiple diseases, mostly concerning colorectal, prostate, breast, lung, and pancreatic cancers, as well as on other pathologies, such as non-Hodgkin’s lymphoma, oral lichen planus, or multiple sclerosis.

## 1. Introduction

### 1.1. Vitamin D in Malignancies

Numerous basic and preclinical studies have suggested a strong relationship between the role of vitamin D in various cancer processes, while different results have been obtained in epidemiological studies and clinical trials [1].

**Figure 1:**
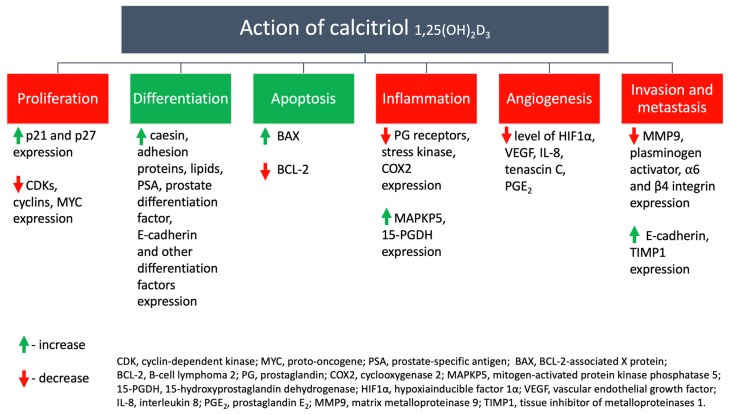
The different effects of calcitriol (the bio-active form of vitamin D) on the human body (based on Feldman et al. 2014 [1], with own modifications).

In the 1980s, several discoveries substantiated the effects of vitamin D on the immune system and important processes in cancer development [2]. In the Suda lab, it was shown that a significant physiological concentration of calcitriol could induce a response in cells unrelated to calcium and phosphate metabolism, as well as stimulate cell maturation of mouse myeloid cells into mature macrophages [2,3]. In addition, a description of an anephtertic patient showed elevated levels of calcitriol and development of secondary hypercalcemia, while macrophages in granulomas in patients with sarcoidosis were found to be responsible for this condition [2,4]. In the study, Provvedini et al. obtained results indicating the presence of vitamin D receptors (VDR) in monocytes and its absence in resting peripheral T and B lymphocytes [5]. At the same time, it was possible to induce *VDR* expression in lymphocytes in response to mitogen or specific antigens, while *VDR* expression was detected in cancer lymphoma cells. Thus, cells can respond to the presence of 1,25(OH)_2_D [2,5], and Colston et al. (1981) proved the anti-cancer effects of calcitriol by inhibiting proliferation of malignant melanoma cells [6]. Until now, the pleiotropic activity of bio-active calcitriol (vitamin D_3_) has been demonstrated in 6 different areas that are important for carcinogenesis (Figure 1). 

Increase in the expression of *p21* and *p27* with a concomitant decrease in cyklin-dependent kinases (*CDks*), cyclins, *MYC* (proto-oncogene) expression inhibits the proliferation of tumor cells. The observed increase in BCL-2-associated X protein (*BAX*) and decrease in *BCL-2* (B-cell lymphoma 2) expression promotes the induction of apoptosis. 

For sporadic colorectal cancer, the presence of calcitriol has an inhibitory effect on ß-catenin transcriptional activity, and thus on the whole activation pathway, which is the most common change in this particular cancer [1,7]. The development of postmenopausal estrogen receptor (ER)+ breast cancer is driven by local synthesis of estrogen, while calcitriol acts as a suppressor of aromatase expression (important in the synthesis of estrogen) [8] and downregulator of ER receptors [1,9]. In addition, the growth of prostate cancer is induced by the activity of androgens (AR). An interaction between the active form of vitamin D and AR, for example, results in the regulation of the AR catabolism [10] and of the expression of the *AR* gene [11] and the *VDR* gene [1,12]. Calcitriol might also have an effect on cancer stem cells, especially those in prostate and breast cancers [1].

**Figure 2:**
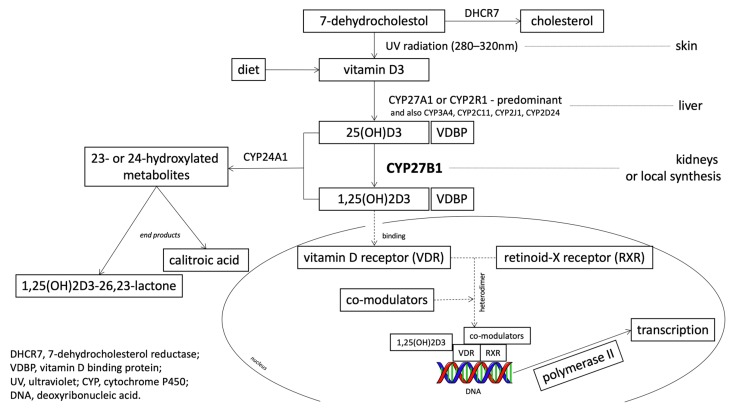
Location of CYP27B1 activity in the metabolic pathway of vitamin D (based on Jenkinson 2019 [13] and Feldman et al. 2014 [1], with own modifications).

All of these described actions of vitamin D pertain to its active form, while 25-hydroxyvitamin D3 1-alpha-hydroxylase (CYP27B1) is the only enzyme capable of transferring the inactive form 25(OH)D into the active 1,25(OH)_2_D form (Figure 2). 

Current literature lacks a summary of existing knowledge about mutations occurring in this *CYP27B1* gene. The first report on linking genetic variation in the key genes of vitamin D metabolism documented the different bone density related to various allelic forms of the vitamin D receptor (*VDR*) [14,15]. The polymorphisms present in *VDR* are the most often studied with respect to the correlation with the presence of specific diseases [16]. The biologically active form of vitamin D has an inhibitory effect on angiogenesis, causes G0/G1 cell cycle arrest, induces apoptosis, increases cell differentiation, and inhibits various signalling pathways in the tumor cell [17]. It is estimated that vitamin D contributes to the expression of 3–5% of genes, including many that are related to cancer [17]. Vitamin D deficiency can affect the development of diseases such as type II diabetes, cardiovascular disease, autoimmune disease, and neoplasms [18]. The plasma level of 25(OH)D is the most commonly used biomarker for assessing vitamin D status from both endogenous synthesis and supplementation [19]. It was assumed that the plasma concentration level of 25(OH)D is an adequate indicator of the supply of vitamin D in the body [20]. 

### 1.2. 25-Hydroxyvitamin D3 1-Alpha-Hydroxylase (CYP27B1): The Gene and the Enzyme

**Figure 3:**
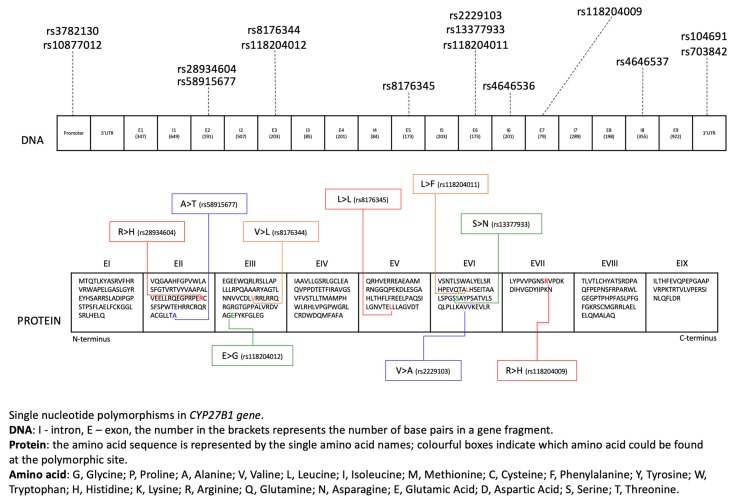
Single nucleotide polymorphisms in amino acid sequences of the *CYP27B1* gene.

It is known that 25-Hydroxyvitamin D3 1-alpha-hydroxylase (*CYP27B1*, cytochrome p450 27B1) encodes the cytochrome P450 enzyme. The location of the gene is at 12q14.1 on the long arm of chromosome 12. Several studies have confirmed that the gene contains 9 exons, but the obtained sum of lengths of exons varies between them [21,22,23]. The enzyme represents a family of 450 cytochromes and is classified as a mitochondrial enzyme. The product of translation is a protein containing approximately 508 amino acids, with an N-terminal mitochondrial signal sequence and a heme binding site [21]. Many single nucleotide polymorphisms (SNPs) were identified in the *CYP27B1* gene, with different effects in amino acid sequence changes (Figure 3).

The role of CYP27B1 is the hydroxylation of 25(OH)D at position C1 and the formation of a biologically active form of vitamin D (the so-called calcitriol). Kidneys are indicated as the main source of the enzyme. There are two independent synthesis sites within this organ: the proximal straight tubules and the proximal convoluted tubules [24,25]. The regulation in the proximal straight tubules occurs under the influence of calcitonin (a hormone produced by thyroid C cells), while in proximal convoluted tubules it is a parathyroid hormone that engages cAMP as a signal transducer.

**Figure 4:**
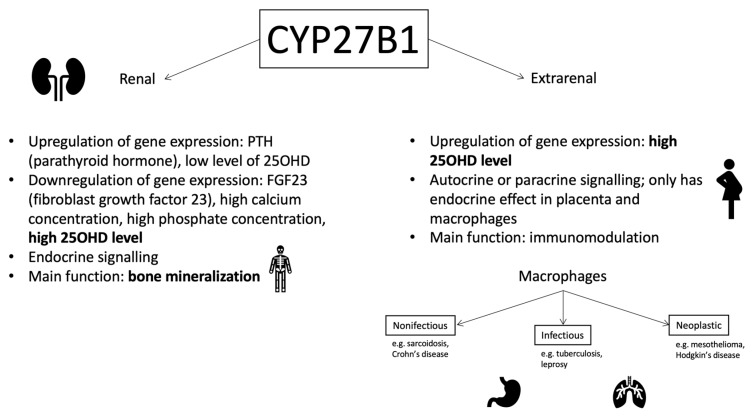
Different roles of CYP27B1 in the human body (based on Adams and Hewison 2012 [26], with own modifications).

Although the enzyme CYP27B1 initially referred only to renal tissues, its presence has been also been determined in numerous other cells from different systems, including dendritic cells, parathyroid cells, osteoblasts, osteoclasts, keratinocyte, mammary epithelial cells, renal tubular cells, pancreatic beta cells, vascular endothelial cells, and prostate epithelial cells [2] (Figure 4). Apart from the activities mentioned above, macrophages also actively express this enzyme, and with granulomata-forming diseases (different infectious, non-infectious, and neoplastic diseases) the production of the enzyme is elevated to a level that it dysregulates calcium homeostasis [26]. 

In addition to macrophages, only the placenta is capable of non-renal 1,25(OH)_2_D synthesis in such large quantities that it causes an increase in its concentration in the blood and has an endocrine effect [26]. However, it is suggested that the role of placental calcitriol is to modulate the fetal immune response, and it is the fetal kidneys that are responsible for the calcium-phosphate metabolism [27].

The regulation of *CYP27B1* expression also involves other mechanisms, depending on its renal or extrarenal origin. In the kidneys, it is controlled by the parathyroid hormone (PTH) and fibroblast growth factor 23 (FGF23), while additionally there is a negative feedback between 25(OH)D and 1,25(OH)_2_D synthesis. Extrarenal *CYP27B1* expression is upregulated by the presence of pro-inflammatory cytokines, such as interleukin 15 (IL-15) or interferon gamma, and is also stimulated by an increased level of 25(OH)D [26].

### 1.3. CYP27B1 and Cancer

The expression of *CYP27B1* and activity of the enzyme in cancerous cells varies depending on the study, either increasing, decreasing, or remaining at the same level [1]. This can be influenced by the grade of tumor, reflecting the abnormality of the tumor cells and tissue, and the probability of growth and spreading behavior of the tumor. Well-differentiated tumors tend to express a higher level of the enzyme than those that are poorly differentiated with an aggressive course of the disease [28].

### 1.4. Colorectal Cancer (CRC)

Most studies tend to conclude that higher levels of vitamin D are negatively correlated with the risk of colorectal neoplasia [29,30,31,32]. By demonstrating that intestinal cells from the large intestine synthesize 1-α-hydroxylase and can convert vitamin D into an active paracrine and autocrine form [33], the influence of the *CYP27B1* polymorphism on the resulting vitamin D level in plasma has become a potential subject of research to determine the risk of the occurrence of colon cancer [34]. It has already been proven that genetic variants occurring within *CYP27B1* increase the risk of colorectal cancer [35]. Alongside the development of the stage of cancer (the appearance of less differentiated cells), decreased expression of the examined gene was observed [36]. It is worth noting that at the very beginning of the tumor transformation, the level of expression is raised compared unchanged neoplastic cells [36]. The effect of vitamin D on the development of colorectal cancer is caused by the inhibition of angiogenesis and activity of the Wnt pathway. Moreover, this pathway also affects the process of angiogenesis and consequently decreases it even more [1,37].

Interestingly, the results indicating the significance of vitamin D were obtained in the winter period, when higher mortality rates occurred after diagnosing the cancer. This period is characterized by vitamin D deficiency compared to individuals diagnosed at the turn of summer and autumn, when higher plasma levels of 25(OH)D were noted [38].

Although a genome-wide association study (GWAS) did not show a relationship between variability within the *CYP27B1* gene and the circulating levels of 25(OH)D [39], two other studies confirmed that the rs10877012 polymorphism is related to the level of 25(OH)D circulating in the blood [40,41]. The results concerning the occurrence and the effects of this polymorphism are contradictory. During vitamin D supplementation, the influence of this polymorphism on the level of any of the vitamin D metabolites could not be demonstrated, and therefore the researchers rejected this hypothesis without examining the vitamin D levels during supplementation [20]. Supplementation of vitamin D in patients with CRC caused an increase in the plasma level of 25(OH)D, however in the case of 1,25(OH)_2_D, this supplementation effect on elevated levels was not observed, which may be caused by the very close mutual control of the level of 1,25(OH)_2_D by both enzymes CYP27B1 and CYP24A1 [20]. Only in the case of hypercalcemia was the calcitriol level increased [42].

Cancers of the proximal and distal parts of the large intestine differ from each other at the molecular level [43]. The proximal part is supplied by the superior mesenteric artery, whereas the distal part is supplied by the inferior mesenteric artery; additionally, lymphatic drainage is carried out by different nodes and the thinner mucous membrane is characteristic of the distal part [44,45,46]. The regulation at the gene level of *CYP27B1* in the mucosa differs between these parts of the intestine [47]. The question arises of whether the risk of cancer varies depending on the location in the large intestine, considering the metabolism of vitamin D [48]. When testing the polymorphism of rs4646536, there was no greater risk to any of the genotypes, while in the same study the interaction with *CYP24A1* (the presence of a TT homozygote at rs4646536 polymorphic site in combination with at least one rare G allele in rs2259735) was shown to reduce the risk of distal colon cancer development (OR = 0.51, 95% CI: 0.33–0.81) [35]. For individuals with at least one rare C allele in rs4646536 and longer exposure to sun-derived UV rays (in the study this was exposure equal to 75 UV index hours), the risk of cancer of the proximal colon cancer decreased (OR = 0.86, 95% CI: 0.74–1.00) [35]. Despite the detection of a statistical dependence, this variant is found in a poorly conserved region and strong linkage disequilibrium occurs between this and another variant occurring in the 5′ promoter region of the *CYP27B1* gene [35]; the functional significance of both variants is unknown, but because of their location they may play a role in transcription or translation [49].

Another study reviewed the impact of polymorphisms on the synthesis of active enzymes in colorectal cancer cells (rs28934604-R107H, rs58915677-A129T, rs8176344-V166L, rs13377933-S356N, rs2229103-V374A) [50]; they all occur within the active domain of the enzyme that contains the porphyrin ring [51]. Apart from rs8176344 polymorphism, all others significantly reduced the enzymatic activity [50]. Among those four polymorphisms, the most significant difference was recorded at rs28934604 and rs13377933. Although the first one is quite rare, it has been thoroughly examined because it is one of the four etiologic factors in rickets development due to the pseudo-deficiency of vitamin D [22]. The next one is a poorly understood polymorphism, whereby its strong influence on the functions of the enzyme means it is a rare genetic variant [50]. Wild type rs58915677 and rs2229103 caused a decrease in CYP27B1 level in this tumor. These are poorly understood polymorphisms and the association of minor allele frequency (MAF) for a specific race has not been established yet, although it was concluded that the rs229103 polymorphism is considered harmful [50,52]. The polymorphism rs8176344 showed increased activity at high doses of 25(OH)D [53]. However, when the substrate concentration remained at a normal level, no increase in synthesis was observed compared to the wild type; such result may indicate a difference in administered doses of vitamin D that should occur in people with various genotypes [50].

The collected data indicate a protective role of calcium from the diet in the development of colorectal cancer [54]. Vitamin D (well-known for its role in maintaining calcium homeostasis) has a binding site within the calcium-sensing receptor (CASR) promoter, which links these two molecules at the molecular level [55]. Potentially the interaction between the variants in *CASR* and in the *CYP27B1* genotypes, which activates the ligand for the *CASR* promoter, could contribute to the etiology of cancer, however this has not been demonstrated yet [55].

As has already been mentioned, vitamin D has protective effects against the development of colon cancer [56,57,58,59,60]. Among many factors, skin color affects the level of this molecule, and thereby this is associated with an increased risk of cancer [61]. The effects of *CYP27B1* polymorphisms on African Americans were analyzed. The study of the two polymorphisms (which in previous studies revealed a connection with vitamin D level [39]), rs10877012 and rs4646537, did not reveal an increased risk either the proximal or the distal colon in this race [62].

Treatment of metastatic colorectal carcinoma is generally based on 5-flurouracil, which significantly improved the efficacy of treatment [63], however to optimize such treatment new drugs are still being searched. So far, only the influence of the rs4588 polymorphism in *VDBP* (vitamin D binding protein) on the chosen treatment has been studied [63]. This may be extended to *CYP27B1* in the near future.

The process of neoplasia within the large intestine is associated with the presence of low levels of 25(OH)D [64,65,66] and 1,25(OH)_2_D [56,67]. In addition to malignant neoplasms, the effect of rs4646536 polymorphism on colorectal adenoma was also investigated, however without success, as it did not affect either 1,25(OH)_2_D or 25(OH)D levels in the blood [68]. Other polymorphisms, such as rs28934604, rs58915677, and rs2229103, were discarded on account of the high monomorphism (most probably due to the suspicion of the pathogenic effect of variation), while rs10877012 (whose effect of vitamin D level in the blood was proven) was not taken into account due to the low MAF value [68].

### 1.5. Prostate Cancer

After the large intestine, the prostate is the next place where calcitriol is synthesized to satisfy the body’s own needs [69].

In vitro, vitamin D affects human prostate cells by acting as a proliferation inhibitory factor [70]. Increased level of *CYP27B1* expression is characteristic for the initial stage of malignant tumors, acting as an inhibitor of the progression of the disease [71,72]. However, other sources report that this phenomenon does not occur when it comes to prostate cancer [70]. This is explained by epigenetic mechanisms, so genetic variability will not impact to a large extend [73]. With the circulating 25(OH)D levels taken into account, the results were inconsistent. Some studies indicated that the level of this molecule diminishes the mortality associated with prostate cancer [74], while another did not find such an association [75], and yet another article found a link with tumor lethality [69].

The study of five polymorphisms within *CYP27B1*, namely rs1048691, rs4646537, rs703842, rs8176345, and rs10877013, did not show an increased risk of developing this cancer in any of the genotypes [76]. The results were obtained basing on the level of 25(OH)D and 1,25(OH)_2_D in serum [76]. Considerations about subsequent polymorphisms yielded results in only one case. For rs3782130, which is evolutionarily non-conserved and located in the 3′UTR, the risk of mortality resulting from prostate cancer was reduced, while rs4646537 did not give such results [71]. However, after repeated comparisons, rs3782130 did not show significance [71]. Interestingly, this polymorphism, along with five others within *VDR* or *CYP24A1*, was included in the SNP panel, which together with the clinical predictors strengthened the sensitivity of the test for 10-year prostate cancer-specific mortality from 91.4% to 94.3% (OR: 2.9, 95% CI: 4.0%, 15.5%, *p* = 0.2) [71].

None of the three examined SNPs (rs10877012, rs8176345, and rs1048691) showed an increased risk of lethal prostate cancer [69].

Further research showed that among African Americans and older men of other races, the prostate cancer mortality rate is increasing because of the amount lowered of absorbed UV waves through the skin [77]. Among the African American representatives, the level of circulating 25(OH)D was two times lower compared to controls [78], which may be suspected to result in a lower protective effect against cancer [79]. Prostate cancer is known to be more common among African Americans [80], partially due to their socio-economic status [81]. A study of this polymorphism group containing rs4646537, rs3782130, rs10877012, and rs703842 did not reveal any association with tumor development [79]. From the group of rs1048691, rs4646537, rs4646536, rs8176345, rs10877012, and rs703842, it was found that rs8176345 was among the 12 most common SNPs in Caucasians and African Americans, which showed statistical significance in this set of SNPs [82]. When investigating the interaction, simultaneously with the SNPs among *CYP17*, *CYP19*, *CYP24A1*, and *CYP1B1* (rs10012, rs17115144, rs1048691, rs11636639, and rs3751592), this polymorphism showed a slight interaction in increasing the risk of prostate cancer development (permutation *p* = 0.04, CVC (cross-validation consistency), 10/10; testing accuracy, 0.60) in African Americans [82].

In general, the association of three variables, the occurrence of a SNP within *CYP27B1* (rs10877012, rs8176345, rs1048691), together with the level of circulating 25(OH)D did not show any relation to the mortality rate among those suffering from cancer [83]. The study of 25(OH)D levels and the correlation with the level of cancer mortality may not reflect the real situation in the prostate, because it is CYP27B1 that determines the bioavailability of the active metabolite of vitamin D [83]. Nevertheless, clinical trials have shown that vitamin D supplementation increased its circulating level, as well as the prostate tissue level [84].

These papers differ greatly in the reported levels of circulating vitamin D and the effect of a particular SNP. A possible explanation could be the differences between the study groups. Vitamin D can positively affect the population in which the PSA (prostate-specific antigen) is monitored with the disease at an early stage and provide therapeutic possibilities among other populations with advanced stages of cancer and restricted therapeutic possibilities [83].

### 1.6. Breast Cancer

The breast tissue is the next place where the CYP27B1 enzyme is present, and thus also the next place where conversion to calcitriol occurs [85]. The results indicate a positive correlation between longer exposure to the sun and reduced breast cancer mortality [86,87], whereas the collected data indicate mixed results when it comes to the effects of diet or supplementation [88,89]. The higher circulating level of 25(OH)D (i.e., above 38.0 ng/mL) in blood reduced the risk of cancer development over the next five years (HR = 0.79; CI: 0.63–0.98) [90]. Epidemiological studies indicated a positive effect for women with postmenopausal cancers. 

The polymorphism rs4646537 was excluded because the MAF was below 5% [91]. The polymorphisms that were selected for this study appeared in earlier papers as factors influencing the occurrence of breast cancer [92]. The polymorphism rs10877012 in another study was taken into account, as only polymorphisms with MAF below two percent were excluded; a study within this polymorphism showed an interaction with 25(OH)D levels in relation to breast cancer development [90]. As the authors point out, this gene may play a role in the prevention of cancer in individual cases, depending on the amount of UV-B radiation and the content of vitamin D in the diet [85]. The study conducted in the Swedish population showed no effect of the selected polymorphism within *CYP27B1* (rs4646537) on risk of cancer, as well as no effect of any SNP within the *VDR, CYP24A1, RXRA* (retinoid X receptor alpha)*, GC/VDBP,* 7-dehydrocholesterol reductase (*DHCR7*)*,* and *CYP2R1* genes (112 SNPs in total) [93].

Two facts need to be considered: about 90% of vitamin D comes from biosynthesis in the body [94] and decreased levels in the case of dark pigmentation drove attention to investigating the occurrence of breast cancer in African Americans. Women with African American roots are at a higher risk of developing breast cancer at a younger age, which will be characterized by greater malignancy. This might be based on the negative status of estrogen receptors or lack of expression of estrogen and progesterone basal receptors [95,96]. The research showed that among African Americans, the level of 25(OH)D was generally lower. The next *CYP27B1* study did not show an increased risk of breast cancer, even after differentiating the neoplasms in those with and without ER expression [97]. This article pointed out that other genes might have more frequent variations typical of Africans, so compiling a low level of 25(OH)D and a particular genotype can predispose people to the development of a specific type of cancer [97].

Primary stage breast cancer is widely treated with third generation aromatase inhibitors (AI) [98], however about a quarter of such patients develop AI-related arthralgia [99], which often terminates the course of therapy [100]. The mechanism of this undesirable effect is not fully understood. One of the suggested reasons is a deficiency of vitamin D, which is very common in postmenopausal women with this cancer [101]. One study examined whether polymorphisms in *CYP27B1* (rs10877012 and rs4646536) contribute to this side effect of the therapy [102]. Both SNPs showed a relationship with the increase in pain at three months after the end of therapy (*p* < 0.05) but they did not act in correcting the false discovery [102]. The interaction of the polymorphism rs3636536 within the *VDR* due to the functional relationship at 3 months after ending the therapy turned out to be borderline significant (*p* = 0.06), while the correlation of pain intensity in rs4646536 with the genotype CC in rs11568820 (*VDR*) proved to be significant (*p* = 0.02) [102]. Subsequent association of the rs4646536 gene variant occurred at rs6163 in *CYP17A1* (*p* = 0.01), and the subsequent assessment of the intensity of pain with the genotype CC in rs6163 proved to be significant for both at 3 (*p* = 0.0006) and 12 (*p* = 0.003) months after ending treatment [102]. Having four adverse alleles within *CYP27B1* and *CYP17A1* increased the risk of AI-related arthralgia five-fold over a 12 months period [102]. In this study, no significant difference in 25(OH)D serum concentrations was noticed, but perhaps even normal circulating levels could not compensate for less functional enzymes in the vitamin D pathway [102].

### 1.7. Lung Cancer

Non-small cell lung cancer (NSCLC) is one of the most common malignancies, contributing to the largest number of deaths among all cancers annually [103]. For lung cancer, a reduced number of cases has been proven under the influence of increased exposure to UVB radiation, and consequently of synthesized vitamin D [104]. Interestingly, at the time of sufficient exposure to solar radiation (in summer and autumn), the number of deaths associated with this cancer decreases [105]. Circulating levels of 25(OH)D may be a prognostic factor for patients with early non-small cell lung cancer [106]. A Finnish female population-based study revealed a protective effect of elevated plasma 1,25(OH)_2_D levels on the development of lung cancer below the age of 50 [107], while a study in the male population showed reduced mortality at an earlier stage of cancer [108,109]. Another study demonstrated the highest *CYP27B1* expression in alveolar macrophages that were present in advanced stages of the disease [110].

Cigarette smoke suppresses two important processes induced by vitamin D, namely NF-κB (nuclear factor-κB) signalling and the production of pro-inflammatory cytokines by activated epithelial cells [111].

The two polymorphisms rs10877012 and rs3782130 did not increase the risk of developing this type of cancer. When smokers and non-smokers were compared between themselves, correlating these polymorphisms with the plasma concentration of 25(OH)D did not show any statistical significance [112]. This is consistent with another GWAS, where there was no association between polymorphism in *CYP27B1* and level of 25(OH)D [39]. However, regardless of the genotype, smokers had a significantly reduced level of 25(OH)D [112]. A study analyzing the same polymorphisms rs10877012 and rs3782130 showed a generally increased risk of lung cancer with the genotype GG at the rs3782130 polymorphic site (adjusted OR = 1.60, 95% CI: 1.09–2.34) [113]. When the population was divided into smokers and non-smokers, results showed a nearly two-fold increase in the risk for smokers, where non-smokers did not show statistical significance (OR = 1.91, 95% CI: 1.04–3.53) [113]. In addition to rs3782130, polymorphisms from the risk group include rs6068816 with homozygote CC (*CYP24A1*), rs4809957 with homozygote AA (*CYP24A1*), and rs7041 with TT homozygote (*GC/VDBP*). The study of multiple polymorphisms revealed that with one allele from these known risk polymorphisms, the chance of developing a cancer increased by 2.5 times on average, with two polymorphisms more than four times, and with three polymorphisms almost six times [113]. Rs3782130 is present within the 3′ end of the promoter and this location may influence the *CYP27B1* gene transcription process, as was demonstrated by the lower gene transcription rate in tumor cells [113]. Elevated *CYP27B1* expression could, thus, act as a prognostic factor for a higher survival rate in patients, especially for the older ones [113]. The explanation of a reduced survival when the GG genotype is present at the rs3782130 polymorphic site could be lower gene expression [113]. The rs3782130 polymorphism is considered to be the nuclear factor 1 binding site, which has a regulatory function in the transcription of many genes [114,115]. The occurrence of a particular genotype may, thus, have a different effect in the regulation of transcription [113].

### 1.8. Pancreatic Cancer

Over 90% of patients survive less than five years after diagnosis [116] and there is an urgent need to determine whether vitamin D plays a role in this cancer, which has a very high mortality rate. As in previously described malignancies, many studies suggest a reduced risk of pancreatic cancer with a high vitamin D status [117]. In areas with higher exposure to sunlight, and thus higher levels of vitamin D biosynthesis, fewer deaths occurred due to pancreatic cancer [118,119,120]. However, contradictory data can be found with regard to serum 25 (OH) D levels, whereby in one study the elevated level indicated a higher risk of pancreatic cancer [121], whereas in another study lower or null risks occurred [122].

These studies included three polymorphisms within *CYP27B1*, of which rs10877013 was of greatest importance, while the others were rs703842 and rs1048691. However, none of them showed statistical significance in the risk of pancreatic cancer or in the 25(OH)D plasma concentration [117]. A Canadian population study within the of polymorphisms rs10877012, rs4646536, and rs703842 did not show an increased risk for the development of pancreatic cancer [116].

### 1.9. Thyroid Cancer

The thyroid is another *CYP27B1* expression site [123]; however, no polymorphism within *CYP27B1* has been studied for any type of thyroid cancer. The existing data are contradictory. The low level of vitamin D was initially associated with an increased risk of thyroid cancer development, however *CYP27B1* expression is increased in the case of thyroid nodular cancer [124]. A study evaluating the expression of *VDR* in thyroid cancer cells of different lines showed that *VDR* expression is at a different level, and a high level does not always provide a positive response to vitamin D therapy [125]. Additionally, it could be assumed that the problem lies within CYP27B1 protein, but the study analyzed the level of CYP27B1 mRNA and showed that among cells more resistant to vitamin D therapy, this level was higher (but still it generally stayed at a lower level) [125]. The level was not elevated in response to therapy, so it was considered unsuitable for determining the sensitivity to activation [125]. This insensitivity to therapy was explained by down-regulation of 1-α-hydroxylase presence or activity [126]. One of the studies showed that patients with thyroid cancer have a reduced level of 1,25(OH)_2_D compared to healthy ones [127]. We have, as yet, no full understanding of what is happening to CYP27B1 in this type of tumor.

### 1.10. Liver Cancer

The contribution of the hepatitis C virus to the development of hepatocellular carcinoma (HCC) is undeniable [128], but the exact mechanism remains unknown. It was found that polymorphisms in genes encoding pro-inflammatory cytokines and ligands of growth factor receptors play an important role in this pathological process [129]. Even though an attempt to link polymorphisms in *CYP27B1* SNPs rs4646536 and rs10877012 with the occurrence of this type of liver cancer in patients with chronic hepatitis C treated with antiviral drugs for direct action showed a lack of statistical significance, these polymorphisms affect the development of HCC [130]. With genotypes CT and TT at the rs4646536 polymorphic site and genotype GG at the rs10877012 polymorphic site, there was no cancer incidence [130].

### 1.11. Non-Hodgkin’s Lymphoma

Although it is a relatively frequent cancer disease, so far only a few risk factors for the development of this disease have been identified, one of which is the protective role of increased sun exposure [131], especially among younger study participants (13–21 years) [132]. However, there is not much data correlating 25(OH)D with the risk of disease, with one study showed no association [133]. Additionally, an analysis of germline variations within *CYP27B1* rs4646537 and rs703842 did not show an increased risk for any of the variants [132].

### 1.12. Other Diseases

For rs10877012 (promoter, C > A), the association between the occurrence of specific alleles and an increased risk of Addison’s autoimmune disease was found (for the C vs A alleles OR = 1.53, 95% Cl 1.07–2.19, *p* = 0.02) [16,134], but simultaneously a reduced susceptibility to development of full-blown hepatitis C occurred (the C allele reduces the long-term antiviral response compared to the A allele, *p* = 0.02) [16,135]. If rs4646536 is present in intron 6, the homozygote TT predestines to developing type I diabetes (OR: 2.14, 95% CI: 1.07–1.36, *p* = 0.01) compared to the CC homozygote (the authors highlight the case of linkage disequilibrium (LD) in rs10877012) [16,136]. The same polymorphism plays a role in the development of congestive heart failure in patients with hypertension and with mainly European roots [16,137]. Having the AC genotype at the rs4646537 polymorphic site and having mainly European ancestors reduces the risk of hypertension in the future [16,137].

### 1.13. Multiple Sclerosis (MS)

The autoimmune background of multiple sclerosis indicates a potentially low level of vitamin D as the main environmental factor affecting the etiopathogenesis of the disease [138]. This hypothesis was based on data on the inverse correlation between the occurrence of MS and the amount of exposure to UVB radiation [139,140]. Laboratory tests on a mouse model with experimental encephalomyelitis (MS model) have shown that vitamin D [141] is vital for the development of this pathology, however one of the components of effective treatment therapy was 1,25(OH)_2_D [142]. The study of the three different polymorphisms (rs118204009, rs118204011, and rs118204012) occurring within *CYP27B1* in patients with multiple sclerosis showed that all were carriers of wild alleles [143] and none of the rare variants were found [143]. In in vitro studies, it was previously demonstrated that all three rare genetic variations within these SNPs lead to a loss of enzyme function and lowering of the level of the active metabolite of vitamin D [143]. In another study, rs118204009 was indicated as a polymorphism associated with the occurrence of MS [144], while in another study due to the very rare occurrence of this polymorphism, it was not confirmed by the lack of people with this rare variant [145]. A meta-analysis focusing only on rs703842 showed that the C allele significantly reduced the risk of multiple sclerosis in Caucasians [146].

### 1.14. Vitamin-D-Dependent Rickets Type 1

Different types of mutations within the *CYP27B1* gene, such as sense-change mutations, insertions, and deletions, lead to rats associated with vitamin-D-dependent rickets type 1, which is a rare autosomal recessive disease [147]. Of these mutations, point mutations have also been identified. Genotyping of two young patients diagnosed with this disease showed that they had two new single nucleotide mutations in the *CYP27B1* gene, marked as H441Y and R459L. This study showed that the first one minimally reduced the activity of the enzyme, while the second mutation reduced it dramatically [148]. The effect of R459L was to prevent CYP27B1 from interacting with adrenodoxin by creating a sulfide bridge, while H441Y inhibited the formation of hydrogen bonds with the same protein [148]. Another study showed a new c.1215 T>C (p.R379R) point variance at the very end of exon 7 in one of seven patients with rickets, which did not change the amino acid in the resulting protein. It is not known whether it affects the splicing process [149]. In the future, newly identified genetic variations may appear in other studies and in the dbSNP (Single Nucleotide Polymorphism Database), with a note that they are associated with the development of this type of the rickets.

### 1.15. Oral Lichen Planus

Oral lichen planus is a disease of unknown etiology, the course of which is characterized by inflammation of the oral mucosa [150]. It is considered to be a cancerous change due to some individuals developing cancer [151]. Due to this characteristic and the combination with the effect of inhibiting carcinogenesis through vitamin D, it is worth looking at the influence of SNP on the etiopathogenesis of this disease. Only one polymorphism rs4646536 was tested within *CYP27B1* and there was no modification of the risk of lichen at any of the genotypes, but this polymorphism was associated with having a denture or artificial jaw [152]. Analysis of the multiloci interactions showed that the model where the polymorphism rs4646536 was present, as well as the polymorphisms in *CYP24A1* (rs22962410), *VDR* (rs4516035 and rs2228570), and *MTHFR* (rs1801133), showed the best testing balancing accuracy and cross-validation consistency [152]. The obtained results of the lack of association of SNP in *CYP27B1* are consistent with those obtained in the study of the risk of developing oral cancer [153].

## 2. Conclusions

The process of carcinogenesis is an extremely complex process that is influenced by numerous genetic and environmental factors. It should be noted that the largest impact of the level of 25(OH)D does not come from genetic factors, but from diet and exposure to sunlight only [154]. If there is a correlation between SNPs and a specific cancer, it can only indicate a moderate risk of disease. The greatest effect of polymorphisms was observed in the case of colon cancer. When it comes to the prostate, there was only one polymorphism rs8176345 noticed in the group of African Americans. In breast cancer, a relationship was observed at rs10877012, whereas in some cases rs4646536 contributed to the occurrence of side effects during the treatment of the pathology. The risk of small-cell lung cancer underwent modifications in the case of rs3782130. The two other polymorphisms rs10877012 and rs4646536 have an impact on the development of hepatocellular carcinoma in HCV patients. There was no evidence of dependence for pancreas, thyroid, or non-Hodgkin’s lymphoma. The important role of vitamin D in the ethiopathogenesis of multiple sclerosis and vitamin-D-dependent rickets type 1 was based on *CYP27B1* polymorphisms. A summary of the role of polymorphisms in *CYP27B1* gene in the occurrence of diseases is provided in Table 1. 

Genotyping of SNPs is an easy, accessible procedure; moreover, it is sufficient to perform it once in a lifetime. Based on the gathered pieces of information, it would be possible to create a panel of SNPs in *CYP27B1* that are associated with malignancies and other chronic diseases, and as a consequence, identify the group with the highest risk of incidence of these diseases. It could thereby contribute to prevention or diagnosis of these illnesses at an early stage.

## Figures and Tables

**Table 1:** *CYP27B1* gene polymorphisms (SNPs) and their roles in disease occurrence.

Single Nucleotide Polymorphism	Location	Description	Frequency	Pathology	Reference
rs10877012	5′ of the gene/promoter region	G > T	T = 0.27826 (34940/125568, TOPMED)	Colorectal cancer—contradictory results Prostate cancer—no association Breast cancer Lung cancer—contradictory results Pancreatic cancer—no association Liver cancer—only in interaction with other polymorphism Addison’s disease Full-blown hepatitis C	[16,39,40,41,69,79,82,83,85,90,112,113,116,130,134,135]
rs4646536	Intron 6	A > G	G = 0.37843 (93102/246024, GnomAD)	Colorectal cancer and adenoma—contradictory results (interactions and exposure to UV rays) Prostate cancer—no association Breast cancer—contribute to side effect of AI therapy Pancreatic cancer—no association Liver cancer—only in interaction with another polymorphism Type 1 diabetes Congestive heart failure Oral lichen planus and oral cancer—no association	[16,35,68,79,82,102,116,130,136,137,152,153]
rs28934604	Exon 2	C > T	None	Colorectal cancer—huge impact on enzymatic activity in cancerous cells	[50]
rs58915677	Exon 2	C > T	T = 0.00020 (34/170818, GnomAD)	Colorectal cancer—impact on enzymatic activity in cancerous cells	[50]
rs8176344	Exon 3	C > G	G = 0.00993 (2404/242112, GnomAD)	Colorectal cancer—impact on enzymatic activity in cancerous cells only in case of high concentration of 25(OH)D	[50]
rs13377933	Exon 6	C > T	None	Colorectal cancer—huge impact on enzymatic activity in cancerous cells	[50]
rs4646537	Intron 8	T > G	G = 0.05028 (6314/125568, TOPMED)	Colorectal cancer—no association Prostate cancer—no association Breast cancer—no association Non-Hodgkin’s lymphoma—no association Hypertension	[16,62,71,79,82,93,132,137]
rs2229103	Exon 6	A > G	G = 0.00001 (2/246262, GnomAD)	Colorectal cancer—impact on enzymatic activity in cancerous cells; this polymorphism is harmful	[50,52]
rs1048691	3′ UTR	C > T	T = 0.26822 (33680/125568, TOPMED)	Prostate cancer—contradictory results Pancreatic cancer—no association	[69,76,82,83,117]
rs703842	3′ UTR	A > G	G = 0.38360 (92103/240104, GnomAD)	Prostate cancer—no association Pancreatic cancer—no association Non-Hodgkin’s Lymphoma—no association Multiple sclerosis	[76,79,82,116,117,132,146]
rs8176345	Exon 5	C > T	T = 0.02302 (5668/246250, GnomAD)	Prostate cancer—contradictory results	[69,76,82,83]
rs10877013	N/A	C > T	T = 0.38135 (88444/231922, GnomAD)	Prostate cancer—no association Pancreatic cancer—no association	[76,117]
rs3782130	5′ of the gene/promoter region	G > C	C = 0.28052 (35224/125568, TOPMED)	Prostate cancer—contradictory results Lung cancer—contradictory results	[71,79,112,113]
rs118204009	Exon 7	C > T	T = 0.00010 (25/246260, GnomAD)	Multiple sclerosis	[143,144]
rs118204011	Exon 6	G > A	None	Multiple sclerosis—no carers of minor alleles	[143]
rs118204012	Exon 3	T > C	C = 0.0009 (91/97256, ExAC)	Multiple sclerosis—no carers of minor alleles	[143]

A, adenine; T, thymine; C, cytosine; G, guanine; TOPMED, Trans-Omics for Precision Medicine; GnomAD, Genome Aggregation Database; ExAC, Exome Aggregation Consortium; UV, ultraviolet; AI, aromatase inhibitor.
